# Facile Semiconductor *p*–*n* Homojunction Nanowires with Strategic *p*-Type Doping Engineering Combined with Surface Reconstruction for Biosensing Applications

**DOI:** 10.1007/s40820-024-01394-5

**Published:** 2024-05-14

**Authors:** Liuan Li, Shi Fang, Wei Chen, Yueyue Li, Mohammad Fazel Vafadar, Danhao Wang, Yang Kang, Xin Liu, Yuanmin Luo, Kun Liang, Yiping Dang, Lei Zhao, Songrui Zhao, Zongzhi Yin, Haiding Sun

**Affiliations:** 1 iGaN Laboratory, School of Microelectronics, University of Science and Technology of China, Hefei, 230026 People’s Republic of China; 2 Department of Obstetrics and Gynecology, The First Affiliated Hospital of Anhui Medical University, No 218 Jixi Road, Hefei, 230022 People’s Republic of China; 3 Department of Electrical and Computer Engineering, McGill University, 3480 University Street, Montreal, QC H3A 0E9 Canada; 4 Union Hospital, Tongji Medical College, Huazhong University of Science and Technology, No 1277 Jiefang Ave., Wuhan, 430022 People’s Republic of China

**Keywords:** *p*–*n* GaN nanowires, Strategic *p*-doping, Surface decoration, Photoelectrochemical sensor, Glucose sensing

## Abstract

Photosensors with versatile functionalities have emerged as a cornerstone for breakthroughs in the future optoelectronic systems across a wide range of applications. In particular, emerging photoelectrochemical (PEC)-type devices have recently attracted extensive interest in liquid-based biosensing applications due to their natural electrolyte-assisted operating characteristics. Herein, a PEC-type photosensor was carefully designed and constructed by employing gallium nitride (GaN) *p*–*n* homojunction semiconductor nanowires on silicon, with the *p*-GaN segment strategically doped and then decorated with cobalt–nickel oxide (CoNiO_x_). Essentially, the *p*–*n* homojunction configuration with facile *p*-doping engineering improves carrier separation efficiency and facilitates carrier transfer to the nanowire surface, while CoNiO_x_ decoration further boosts PEC reaction activity and carrier dynamics at the nanowire/electrolyte interface. Consequently, the constructed photosensor achieves a high responsivity of 247.8 mA W^−1^ while simultaneously exhibiting excellent operating stability. Strikingly, based on the remarkable stability and high responsivity of the device, a glucose sensing system was established with a demonstration of glucose level determination in real human serum. This work offers a feasible and universal approach in the pursuit of high-performance bio-related sensing applications via a rational design of PEC devices in the form of nanostructured architecture with strategic doping engineering.

**Supplementary Information:**

The online version contains supplementary material available at 10.1007/s40820-024-01394-5.

## Highlights

- A novel photoelectrochemical (PEC) photosensor composed of GaN nanowire-on-Si platform demonstrates record-high responsivity of 247.8 mA W^−1^ with ultra-stable operation characteristics.
- Strategic internal and external band structure engineering of semiconductor nanowires promotes efficient PEC reaction via controlling carrier dynamics while preserving nanowires from material degradation.
- The glucose sensing system is constructed to successfully analyze blood glucose levels in real human serum samples, featuring a high sensitivity of 0.173 µA µM^−1^ cm^−2^ and a low detection limit of 0.07 µM.

**Supplementary Information:**

The online version contains supplementary material available at 10.1007/s40820-024-01394-5.

## Graphical abstract

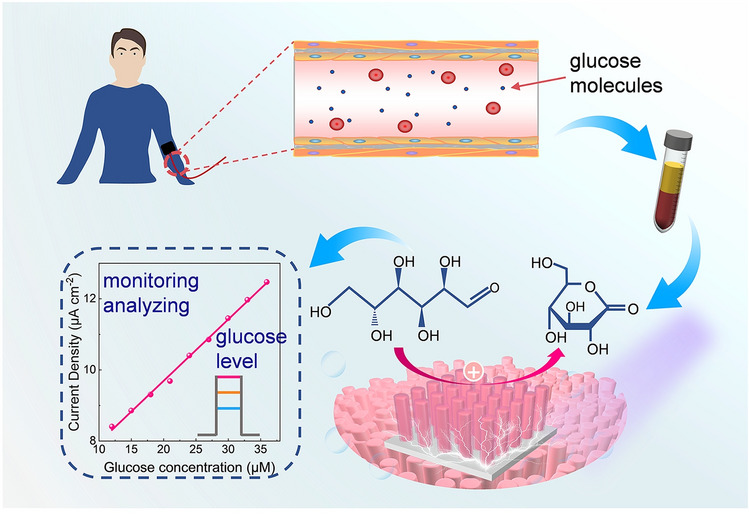

## Introduction

Photosensors, which convert incident light signals into electrical signals, play a significant role in constructing effective optoelectronic integrated systems [1, 2]. Particularly, the emerging sensors with versatile functionalities that can directly operate in harsh environments, e.g., in liquid conditions without sophisticated device packaging, are highly in demand [3, 4]. Photoelectrochemical (PEC)-type devices, based on their unique working principles that combine semiconductor physics with the chemical reaction process, have attracted extensive interest due to their simple fabrication, low cost, self-powered, and appealing electrolyte-assisted device properties [2, 4, 5]. Essentially, due to their electrolyte-assisted operating characteristics, the operation principle of PEC-type devices not only follows the conventional carrier generation, separation, and migration processes in semiconductors but also encompasses a unique electrochemical procedure that involves the redox reaction at the semiconductor/electrolyte interface, providing us more freedom to regulate their photoresponse behavior [6, 7]. Thus far, the PEC-type devices have unleashed their potential in the fields of underwater optical communication [8], optoelectronic logic [9], artificial vision [2], automatic imaging [10], energy conversion catalysis [11], etc. Additionally, the unique electrolyte-assisted operation might also enable PEC-type devices for possible liquid-based biosensing, including glucose detection, DNA detection, and cell detection [12–14]. Similar to other analytical approaches or techniques such as electrochemical bioanalysis, emerging PEC-type photosensors, which naturally operate in a liquid environment, allow easy linkage with the biological world [15]. In addition, differing from electrochemical methods that require an external electrical power source to generate electrical detection signals, PEC-type photosensors, which separate the electrical detection signals from optical excitation sources, could contribute to negligible background signals and reduce possible interference from electroactive substances, making them an ideal sensor architecture for biosensing applications [16].

In the exploration of efficient biosensors, responsivity and stability stand as key characteristics, exerting a direct influence on the overall sensing performance in various application scenarios [17]. Concretely, high responsivity ensures that sensors can acutely capture the change of target substance, while high stability guarantees that sensors can operate continually and reliably in complex electrolyte-related working conditions [18, 19]. However, how to achieve high responsivity and high stability in PEC-type photosensors remains a challenge to ensure optimal sensing performance [13, 16]. Essentially, the performance of PEC-type photosensors depends on the charge carrier transport characteristics in three manners: (1) within the semiconductor, (2) at the semiconductor/electrolyte interface, and (3) in surface chemical reactions, which offers us extreme flexibility to tune and optimize their performance via physical and chemical processes [4–6]. To date, based on these three charge transport characteristics, various studies have been carried out to intentionally tune their photoresponse performance in the pursuit of bio-related sensing applications. For instance, by designing a dual Z-scheme BiVO_4_@Ni-ZnIn_2_S_4_/Bi_2_S_3_ heterojunction, the effective separation of the internal photocarrier accelerated the migration of electrons within the semiconductor, and the responsivity of the photoanode was enhanced, enabling ultrasensitive detection of ofloxacin [20]. Additionally, the surface decoration of CsPbBr_3_ photoanodes with reduced graphene oxides reduced interfacial charge transfer resistance and acted as passivation layers, improving operational stability and enabling mycotoxin detection [21]. Furthermore, the modification of CdS nanorods photoanode and CuInS_2_ nanoflowers photocathode with Fe single-atom catalysts improved their surface redox reaction activity, resulting in enhanced responsivity and achieving highly sensitive and selective uric acid detection [22]. Inspired by these exciting achievements, the unique operation characteristics of internal and external carrier transport coupled with surface reactions offer abundant opportunities in the pursuit of PEC-type bio-photosensors with superior performance, e.g., high responsivity and stability.

In this work, we designed and fabricated a PEC-type photosensor by employing gallium nitride (GaN) *p*–*n* homojunction nanowires with *p*-GaN segment strategic doping, and then, the nanowire surface was decorated with cobalt–nickel oxides (CoNiO_x_). Intriguingly, under light illumination, the *p*–*n* homojunction served as a "holes pump," effectively pumping photogenerated holes to the nanowire surface. Compared to pure *n*-GaN nanowires, the photovoltage of *p*–*n* GaN nanowires increases by 172%, suggesting a significant improvement of internal carrier separation efficiency. Furthermore, the hole barrier at the *p*-GaN/electrolyte interface was minimized by strategic doping of *p*-GaN segment to promote the migration of photogenerated holes to the electrolyte. Thereafter, to further improve the carrier migration process, a rational surface decoration of cobalt–nickel oxide (CoNiO_x_) was applied, forming a *p*–*n* GaN/CoNiO_x_ photoelectrode. Strikingly, we achieved a remarkably high responsivity of 247.8 mA W^−1^ and demonstrated excellent stability of the sensor, exhibiting negligible attenuation during 27.5 h of the stability test. Eventually, due to its remarkable stability and high responsivity, along with a particular aqueous operating environment, we successfully constructed a glucose sensing system with excellent linear response and selectivity, and demonstrated glucose level determination in real human serum. This study provides us with a simple and universal route to unleash the full potential of PEC devices for future advanced biosensing applications.

## Experimental Section

### Growth of the GaN Nanowires

GaN nanowire arrays were grown on planar Si (111) substrates using plasma-assisted molecular beam epitaxy (MBE). The process began with annealing the Si (111) substrates to eliminate any residual oxide. For *n*-type and *p*-type doping, Si and Mg were employed, respectively. The Mg-doping concentration was varied by regulating the Mg effusion cell temperatures (*T*_Mg_). Samples *p*–*n* GaN, *p*^+^–*n* GaN, and *p*^++^–*n* GaN nanowires correspond to Mg fluxes in the range of 1 × 10^–10^–1 × 10^–9^ Torr.

### Photodeposition of CoNiO_x_

The *p–n* GaN nanowires sample was first cleaned with acetone and deionized water to eliminate organic contaminants before photodeposition. Subsequently, the cleaned sample was immersed in a solution containing 8 mL of deionized water, 2 mL of NaIO_3_ solution (0.2 M L^−1^), and 30 µL of CoNO_3_ (0.2 M L^−1^), and 30 µL of NiNO_3_ (0.2 M L^−1^) solutions. The sample was then illuminated for 30 min using a Hg lamp (Tanon UV-100).

### Fabrication of the Photoelectrode

Initially, the as-grown GaN nanowire samples were divided into multiple pieces of the desired dimensions. To establish ohmic contacts, an In–Ga eutectic alloy (Alfa Aesar) was applied to the Si substrate following the removal of the SiO_2_ layer. The coated region was then connected to a copper sheet using silver paste (SPI Supplies). Finally, epoxy was employed to encapsulate the nanowires for insulation and packaging, leaving the front-facing nanowires exposed for photodetection.

### Photoelectrochemical Measurements

The as-fabricated photoelectrode, counter electrode (Pt mesh), and reference electrode (Ag/AgCl) were immersed in an electrolyte solution of 0.01 M PBS. The incident light for the measurements was generated by LEDs with wavelengths ranging from 265 to 365 nm, and the corresponding light intensities were calibrated using an optical power meter (Newport Model No. 2936 R). For the demonstration of glucose sensing, deionized water, and a glucose standard solution were chosen as the electrolytes. The corresponding photoresponse performance of our devices was evaluated by an electrochemical workstation (CHI 660E). The corresponding average photocurrent was obtained by integrating the current over time.

### Material Characterization

The morphologies of the GaN nanowire samples were characterized using a scanning electron microscope (Hitachi SU8220 system, operating at 3 kV) and a transmission electron microscope (JEOL-2100F system, operating at 200 kV). Elemental mapping via energy-dispersive X-ray spectroscopy was performed using a 26FEI Talos F200X device at 200 kV. High-resolution transmission electron microscopy (HRTEM) results were obtained using a JEM-ARM 200 F instrument at 200 kV. X-ray photoelectron spectroscopy (XPS) measurements were conducted on a Thermo Scientific K-Alpha XPS instrument equipped with an Al Kα source (*hν* = 1253.6 eV), operating at 15 kV. TRPL spectra were measured by the TPL-300 (Time-Tech Spectra) with a 230-nm pulsed laser. Kelvin probe force microscopy (KPFM) measurements were conducted using an atomic force microscope (AFM) (NTEGRA SPECTRA, NT-MDT). The probe employed in the measurements is a PtIr-coated tip. Monochromatic lights were provided by a xenon lamp equipped with a monochromator (IHR320, Horiba JY). The detailed test method followed the previous work [23].

## Results and Discussion

### Structural Design of GaN Nanowires with *p*–*n* Homojunction via Strategic *p*-type Doping Engineering

**Fig. 1:**
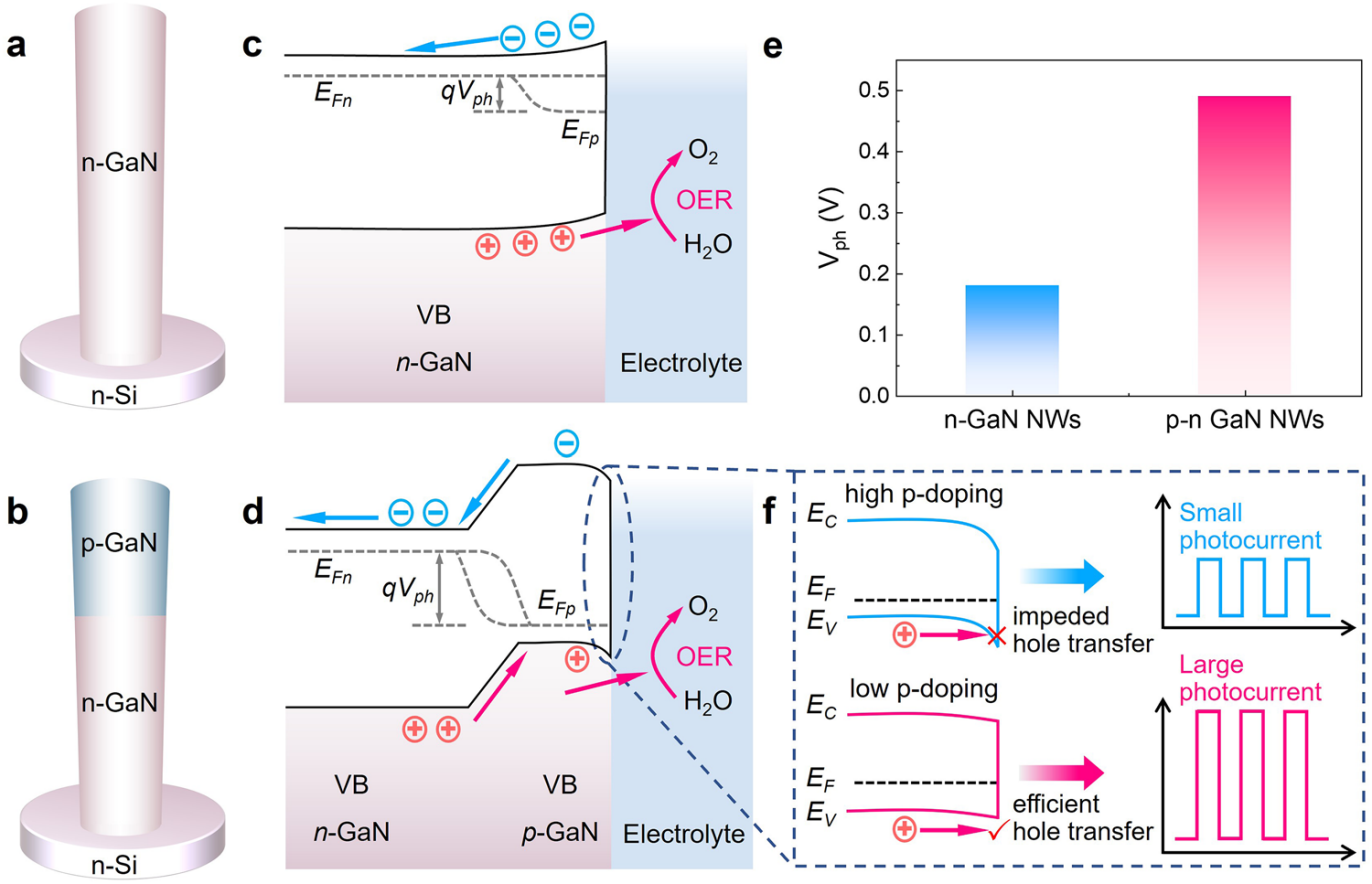
Band structure design of GaN nanowire. Schematic of **a** *n*-GaN nanowire and **b** *p*–*n* GaN nanowire structure. Band diagrams of **c** *n*-GaN and **d** *p*–*n* GaN nanowire in contact with the electrolytes under illumination. The *E*_*Fn*_ and *E*_*Fp*_ are the electron and hole quasi-Fermi levels, respectively. The *V*_*ph*_ represents the photovoltage generated in the nanowires. **e** Comparison of photovoltages of *n*-GaN and *p*–*n* GaN nanowires. Data are extracted from Fig. S1c. **f** Relationship between the *p*-GaN segment surface band bending and the resulting photocurrent

The construction of a high responsivity and stable photoanode is crucial in the pursuit of PEC-type photosensors across a wide range of applications [24]. The *n*-type semiconductors are usually used as photoanodes in PEC-type photosensors, where the photodetection process encompasses charge generation, separation, and charge transfer in the PEC reactions. Specifically, as shown in Fig. 1a and c, when the *n*-GaN nanowire is in contact with the electrolyte, the surface band bends upward at equilibrium [25]. Upon illumination, photogenerated holes migrate toward the nanowire/electrolyte interface, driving the water oxidation reaction (i.e., oxygen evolution reaction, OER). Electrons migrate to the external circuit, finally contributing to the generation of photocurrent. From the perspective of semiconductor band structure engineering, the band bending degree of the *n*-GaN surface determines the carrier separation capability of the whole nanowire [26]. However, the carrier separation within mono-doped (e.g., *n*-type) semiconductors is usually inefficient, leading to limited overall device efficiency [27]. Here, to address this issue, we propose and design a GaN *p*–*n* junction that can pump photogenerated holes transporting toward the nanowire surface under light illumination. Specifically, as shown in Fig. 1b, a GaN *p*–*n* junction can be formed by *p*-type doping for the top segment of the nanowire while maintaining the bottom segment as *n*-type doping. As depicted in Fig. 1d, the formation of the *p*–*n* junction within the nanowire could generate a significant built-in electric field, leading to a large band bending at the *p*-GaN/*n*-GaN interface, which acts as a strong driving force for the separation of photogenerated carriers [26].

To verify the above design, we conducted open-circuit potential (OCP) tests to investigate free carrier dynamics and measure the photovoltage of the *n*-GaN and *p*–*n* GaN nanowires, which were grown under the same conditions except for the *p*-doping of the upper segment of *p*–*n* GaN nanowires (Fig. S1a and b). The difference between OCP under dark/light conditions (ΔOCP) is usually used to characterize the photovoltage (*V*_ph_). As depicted in Fig. S1c, the OCP of the *n*-GaN nanowires shifts to negative potentials under illumination, confirming its electron-conducting *n*-type properties [28]. Similarly, the OCP of the *p*–*n* GaN nanowires also exhibits a negative shift, indicating that the dominant holes migrate toward the surface in the *p*–*n* GaN nanowires, consistent with that of the *n*-GaN nanowires. Importantly, as shown in Fig. 1e, the measured photovoltage of *p*–*n* GaN nanowires (0.49 V) increases by 172% compared to that of *n*-GaN nanowires (0.18 V), consistent with the band design in Fig. 1a–d. Essentially, the *p*–*n* GaN nanowires exhibit a larger photovoltage compared to *n*-GaN nanowires, contributing to a stronger driving force for carrier separation and subsequent photocurrent generation.

As illustrated above, by introducing the *p*-GaN segment to form a *p*–*n* junction, the photovoltage can be prominently improved with the photogenerated holes effectively pumped toward the nanowire surface. However, the downward surface band bending of the *p*-GaN segment notably forms a potential barrier that might hinder the migration of photogenerated holes toward the electrolyte [29]. Meanwhile, since the downward surface band bending of the *p*-GaN segment in contact with the electrolyte might increase with the rise of the *p*-doping concentration level [30], we attempted to employ a doping modulation scheme to optimize the surface band structure of the *p*-GaN segment, trying to maximize the hole migration efficiency to the electrolyte. As illustrated in Fig. 1f, when a high *p*-doping concentration is applied, the significant downward bending of the surface band may create a high potential barrier, impeding the migration of photogenerated holes to the electrolyte. This might result in a small photocurrent. Conversely, at lower *p*-doping levels, the potential barrier may be substantially reduced due to a moderate band bending, allowing photogenerated holes to effectively migrate to the electrolyte and then engage in reactions, thereby generating a larger photocurrent.

### Surface Band Structure Characterization of GaN *p*–*n* Nanowires with Different *p*-type Doping

To verify the influence of *p*-doping on the surface band of the GaN nanowire and its corresponding photoresponse, three types of *p*–*n* GaN nanowires were grown on silicon substrates using the molecular beam epitaxy (MBE) technique, with varied *p*-doping levels by employing different concentrations of *p*-type dopants during the growth of the *p*-GaN segment. Si and Mg were employed as donor and acceptor dopants, respectively, for *n*-type and *p*-type doping (details see Experimental Section). The *p*–*n* GaN nanowires were denoted as *p–n* GaN, *p*^+^–*n* GaN, and *p*^++^–*n* GaN nanowires, corresponding to the nanowires with incremental Mg-doping concentrations.

**Fig. 2:**
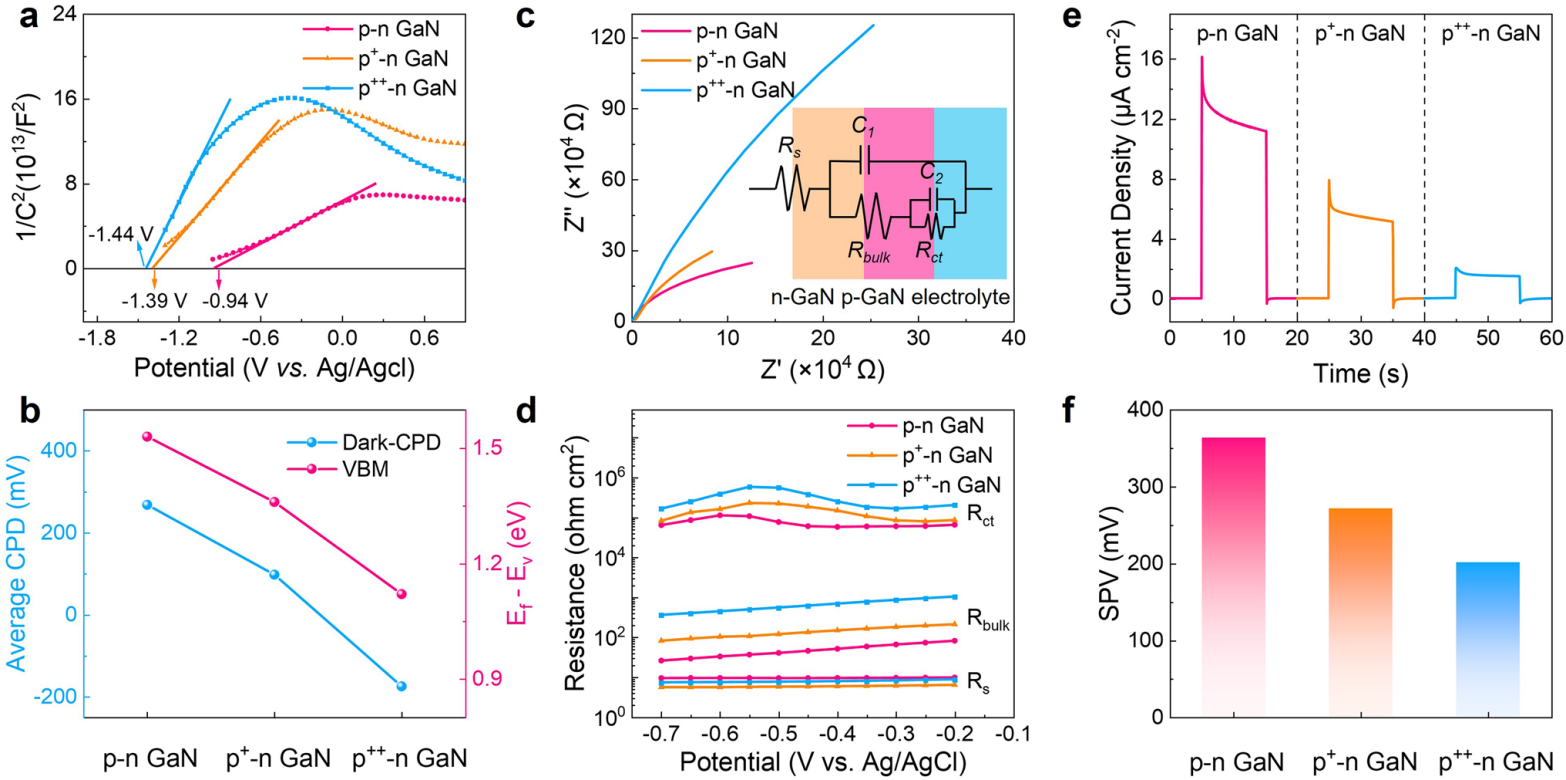
Surface band characterization and investigation of the charge transfer properties. **a** Mott–Schottky plots measured under dark conditions. **b** Surface potential measured by KPFM shows the average CPD values, and XPS valence spectra show the relative location of the valence band maximum to the Fermi level. **c** EIS plots measured at 340 nm with a light intensity of 0.1 mW cm^−2^ (0 V vs. Ag/AgCl). **d** The *R*_ct_, *R*_bulk_, and *R*_s_ fitted values extracted from EIS plots (measured under different applied biases). **e** Photocurrents measured at 340 nm with light intensity of 0.1 mW cm^−2^. **f** SPV measured by KPFM under 340-nm irradiation

Scanning electron microscope (SEM) images of the *p*–*n* GaN nanowires are shown in Fig. S2. These images display a uniform distribution of the nanowires on Si substrate, exhibiting nearly consistent diameters and lengths of the three types of nanowires with different Mg-doping concentrations, and their filling factors are very close at 0.76, 0.77, and 0.80 for the *p*–*n* GaN, *p*^+^–*n* GaN, and *p*^++^–*n* GaN nanowires, respectively. To confirm the formation of *p*–*n* GaN nanowires with varied *p*-doping levels and assess the impact of different *p*-doping levels on the surface band bending, we conducted Mott–Schottky tests [31]. As shown in Fig. 2a, the Mott–Schottky curves reveal a similar inverted “V-shape” with distinct slopes. The positive slope of the curve corresponds to the *n*-type conductivity of the material, while the negative slope indicates the presence of *p*-GaN [32]. The Mott–Schottky diagram provides valuable information by analyzing the linear region of the fitted curve, where the slope reflects the relative magnitude of the donor concentration, and the intercept represents the flat-band potential [33]. The magnitude of the positive slope in the Mott–Schottky curves directly reflects the relative donor concentration in the material, with smaller slope values indicating higher donor concentrations. As a result, the donor concentration is the highest in *p*–*n* GaN nanowires, while *p*^++^–*n* GaN nanowires have the lowest donor concentration. This can be attributed to the compensation of the background *n*-type dopants by the Mg acceptor, and the compensation effect becomes larger with increasing *p*-doping concentration [25]. Significantly, the flat-band potential of *p*–*n* GaN nanowires (− 0.94 V vs. Ag/AgCl) exhibits a positive shift relative to *p*^+^–*n* GaN (− 1.39 V vs. Ag/AgCl) and *p*^++^–*n* GaN nanowires (− 1.44 V vs. Ag/AgCl), indicating a decrease in the bending of *p*-GaN segment band edges [34].

To further investigate the impact of different *p*-doping levels on the surface band bending, we carried out contact potential difference (CPD) measurements using Kelvin probe force microscopy (KPFM) in air to directly probe the work function level. Figure S3a–c shows the surface potential scans in a 2 × 2 μm^2^ region of three types of *p*–*n* GaN nanowires under dark conditions. The average CPD values for *p*–*n* GaN, *p*^+^–*n* GaN, and *p*^++^–*n* GaN nanowires are 268.1, 97.6, and − 175.1 mV, respectively, as depicted in Fig. 2b (extracted from Fig. S3a–c). Here, the CPD is defined as (*Φ*_tip_ − *Φ*_surface_)/ − *e*, where *Φ*_tip_ and *Φ*_surface_ represent the work functions of the Kelvin probe tip and the nanowire surface, respectively, and *e* denotes the elementary charge [35]. The *Φ*_surface_ can be calculated by the function: *Φ*_surface_ = *eV*_CPD_ + *Φ*_tip_. As the *p*-doping concentration rises, the work function of the *p*–*n* GaN nanowires progressively increases, resulting in a closer alignment of the Fermi level with the valence band. Furthermore, to characterize the location of the valence band maximum relative to the Fermi level, XPS was employed to measure the valence band spectra of the nanowires, and the corresponding results are depicted in Fig. S4. In Fig. 2b, the relative positions of the valence band maxima in *p*–*n* GaN, *p*^+^–*n* GaN, and *p*^++^–*n* GaN nanowires were determined as 1.53, 1.36, and 1.12 eV, respectively, by extrapolating the linear region of the XPS valence band spectra in Fig. S4. Based on the analysis of CPD and XPS valence band spectra, the surface Fermi level of the *p*-GaN segment decreases as the *p*-doping concentration rises, causing an increase in the downward band bending at equilibrium in contact with the electrolyte, which is consistent with the Mott–Schottky results. As mentioned above, the downward bending of the surface band forms a potential barrier that hinders the migration of photogenerated holes to the electrolyte. Hence, by reducing the surface band bending at the *p*-GaN segment, it might decrease the hole transport resistance and thereby enhance the photoresponse.

Then, electrochemical impedance spectroscopy (EIS) is used to investigate the electrical properties of the photoelectrode, which reflect the transfer efficiency of charge carriers in the photoelectrode. The previous research has consistently demonstrated that a smaller arc radius in the Nyquist plots is indicative of a lower charge transfer resistance (*R*_ct_) within the photoelectrode [23]. Therefore, we conducted EIS tests under operating conditions (0 V vs. Ag/AgCl, 340-nm light irradiation). The *p*–*n* GaN nanowires exhibit the smallest semicircle diameter (Fig. 2c), confirming the presence of a minimal charge transfer barrier. Furthermore, tests were conducted at various operating voltages and fitted using the equivalent circuit illustrated in the inset of Fig. 2c. The fitted parameters are presented in Fig. 2d. *R*_s_, *R*_bulk_, and *R*_ct_ correspond to the solution resistance, bulk resistance of the material, and charge transfer resistance, respectively. The *R*_s_ values of the three types of *p*–*n* GaN nanowires are nearly identical, indicating the validity of the fitting results. The *R*_bulk_ shows a tendency to increase with the rise of *p*-doping concentration. This is reasonable because the effective donor concentration of nanowires decreases with increasing *p*-doping concentration, leading to overall conductivity decreases [36], which is consistent with the Mott–Schottky results. Importantly, the *R*_ct_ values increase with the rise of *p*-doping concentration. This result verifies that moderate downward surface band bending can reduce the charge transfer resistance, facilitating the migration of photogenerated holes to the electrolyte. Notably, the relations of the resistances among the three photoelectrodes are consistent under different voltages.

To further verify the photoresponse determined by the different surface bands, we evaluated the photocurrents of the photoelectrodes through *I*-*t* tests, as illustrated in Fig. 2e. The photocurrent decreases with increasing *p*-doping concentration, confirming that the severe surface band bending hinders the transfer of holes into the electrolyte, leading to a small photoresponse. Furthermore, we conducted surface photovoltage (SPV) tests using KPFM in air to directly examine the carrier migration behavior. Typically, SPV measurements enable the assessment of the migration capability of photogenerated carriers toward the nanowire surface, and a positive SPV indicates the dominant migration of photogenerated holes toward the nanowire surface [5]. Figure S3 displays the CPD mappings under both dark and 340-nm light irradiation. The SPV is calculated as the difference between the average CPD values measured under illuminated and dark conditions. The SPV value, extracted from Fig. S3 and displayed in Fig. 2f, decreases with increasing *p*-doping concentration. Thus, the SPV results are consistent with the *i-t* results, further supporting that moderate surface band bending promotes the migration of photogenerated holes to the nanowire surface, consequently leading to a high photoresponse.

### Structural Characterization of the CoNiO_x_-Decorated *p*–*n* GaN Nanowires

**Fig. 3:**
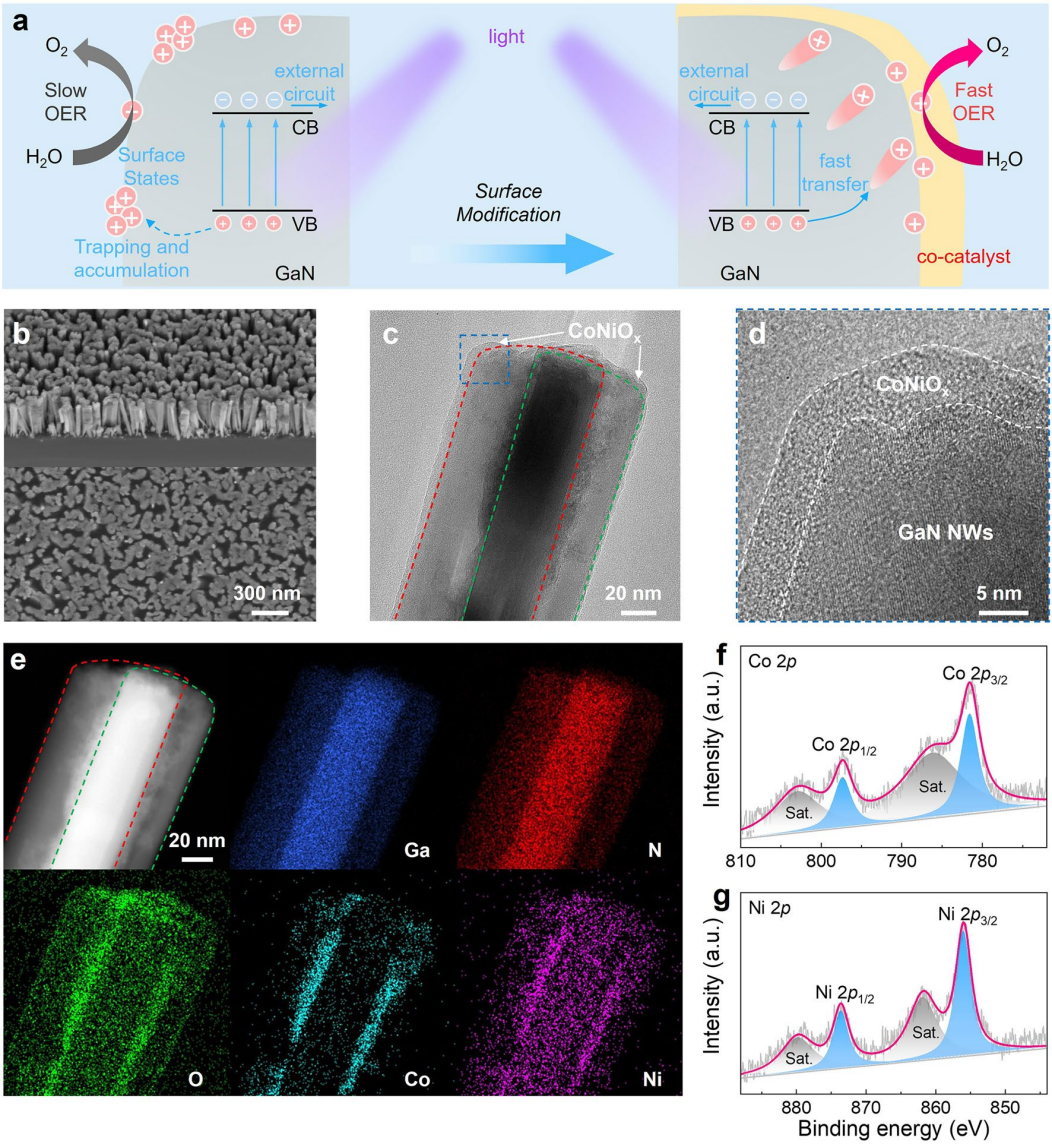
Surface modification and material characterizations. **a** Schematics of the carrier dynamics of the pristine GaN and surface-modified GaN nanowires. **b** 30°-tilted and top-view SEM images of the *p*–*n* GaN/CoNiO_x_ nanowires. **c** TEM image of the *p*–*n* GaN/CoNiO_x_ nanowires. **d** The enlarged image of the blue-outlined area in (**c**). **e** Corresponding EDS elemental mapping of the *p*–*n* GaN/CoNiO_x_ nanowires. XPS spectra for **f** Co 2*p* and **g** Ni 2*p* of *p*–*n* GaN/CoNiO_x_ nanowires

Based on the preceding discussion, we have confirmed that a rational design of *p*–*n* junctions with proper *p*-doping promotes the migration of holes to the nanowire surface while optimizing the transfer of holes into the electrolyte, thereby enhancing the overall performance of the photoelectrode. However, as shown in Fig. 3a, the previous investigations have revealed that the surface states of pristine GaN nanowires can trap photogenerated carriers, thereby impeding surface photoelectrochemical reactions [37]. Additionally, the scarcity of active sites on the pristine GaN surface imposes limitations on the surface reaction rate, hindering the rapid consumption of the photogenerated carriers [38]. Consequently, carrier accumulation on the nanowire surface may compromise device performance [24]. As shown in Fig. S5, the as-constructed *p*–*n* GaN photoelectrode was tested under chopped light. During the test, the photocurrent exhibits a gradual decline, ultimately decreasing by 40.5% after 6200 s of continuous testing, suggesting a persistent deterioration in device performance. To improve the consumption of photogenerated carriers and enhance the stability of the device during long-term operation, surface modification for nanowires is highly desirable. The CoNiO_x_ is a bimetallic oxide known for its exceptional OER catalytic activity and is commonly employed as a hole transport layer to effectively enhance carrier extraction [39]. Herein, CoNiO_x_ co-catalyst was deposited on the *p*–*n* GaN nanowires by using a photodeposition process (details see Experimental Section).

The morphology of the surface-modified *p*–*n* GaN nanowires was analyzed using SEM. Figure 3b shows that the nanowire array remains intact after surface modification. Transmission electron microscopy (TEM) was then employed to further characterize the distribution and microstructure of the surface-modified nanowires. As depicted in Fig. 3c, two overlapped nanowires are discernible, with their contours delineated by red and green dotted lines, respectively. The high-resolution transmission electron microscopy (HRTEM) image (Figs. 3d and S6) demonstrates the presence of an amorphous layer enveloping the nanowires, clearly outlined by the white dotted lines. The energy-dispersive spectroscopy (EDS) elemental mapping (Fig. 3e) performed in the corresponding region illustrates the spatial distribution of Ga, N, O, Co, and Ni elements. These results display the distribution of Co, Ni, and O elements around each nanowire, indicating the presence of CoNiO_x_ species on the surface of nanowires. Furthermore, the chemical state of the decorated Co and Ni species was characterized using XPS. The Co 2*p* spectrum (Fig. 3f) and Ni 2*p* spectrum (Fig. 3g) exhibit characteristic doublets consisting of 2*p*_3/2_ and 2*p*_1/2_ peaks due to spin–orbit coupling, along with shakeup satellite peaks. The characteristic peaks of Co 2*p*_3/2_ and Co 2*p*_1/2_, observed at 781.5 and 797.3 eV in Fig. 3f, respectively, along with their respective satellite peaks (786.1/802.9 eV), indicate the presence of Co^2+^ species [40]. Similarly, Ni 2*p*_3/2_ and Ni 2*p*_1/2_, observed at 855.9 and 873.6 eV in Fig. 3g, respectively, along with their respective satellite peaks (861.8/879.8 eV), confirm the presence of Ni^2+^ species [41]. Combined with the above analysis, we confirmed the successful coating of *p*–*n* GaN nanowires with an amorphous layer of Co^2+^Ni^2+^ oxides (referred to as *p*–*n* GaN/CoNiO_x_ nanowires).

### Photoresponse Behavior Evaluation of *p*–*n* GaN/CoNiO_x_ Nanowires and the Underlying Working Mechanism

**Fig. 4:**
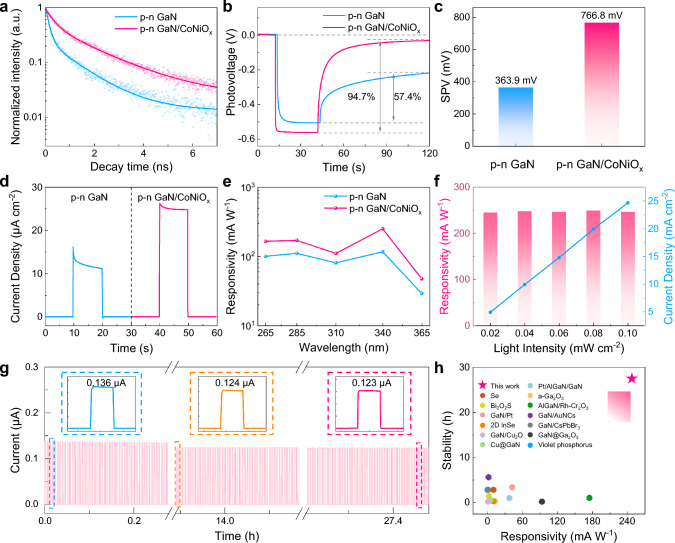
Investigation of photogenerated carrier dynamics and evaluation of photoresponse. **a** TRPL curves of the *p*–*n* GaN and *p*–*n* GaN/CoNiO_x_ nanowires. **b** Open-circuit potential measurements at 340 nm with a light intensity of 0.1 mW cm^−2^. **c** SPV measured under 340-nm irradiation. **d** Photocurrents measured at 340 nm with a light intensity of 0.1 mW cm^−2^. **e** Comparison of the spectral response of the *p*–*n* GaN and *p*–*n* GaN/CoNiO_x_ photoelectrodes. **f** Responsivity and photocurrent density of the *p*–*n* GaN/CoNiO_x_ photoelectrode at 340 nm with various light intensities. **g** Continuous on/off cycle test (27.5 h) of the *p*–*n* GaN/CoNiO_x_ photoelectrode. **h** Comparison of the responsivity and stability of previously reported PEC-type photosensors and this work

To investigate the impact of CoNiO_x_ on photogenerated carrier dynamics, we conducted time-resolved photoluminescence (TRPL) spectroscopy on both *p*–*n* GaN and *p*–*n* GaN/CoNiO_x_ nanowires (Fig. 4a). The emission decay curves were fitted with double-exponential decay functions [42], and the detailed parameters for TRPL lifetime are found in Table S1. The average decay time constant (*τ*_ave_) of the pristine *p*–*n* GaN nanowires is 0.387 ns, while the *p–n* GaN/CoNiO_x_ nanowires demonstrate a longer *τ*_ave_ of 0.875 ns, suggesting a slower photoluminescence decay rate compared to the pristine *p*–*n* GaN nanowires. This implies that CoNiO_x_ decoration suppresses photogenerated carrier recombination and enhances charge separation which might be attributed to the passivation of surface trap states [43]. Furthermore, we conducted OCP tests of *p*–*n* GaN nanowires and *p*–*n* GaN/CoNiO_x_ nanowires (Fig. 4b). Remarkably, due to a slow relaxation process, the OCP of *p*–*n* GaN nanowires exhibits a slow recovery process with a recovery rate of 57.4%, confirming the trapping effect of surface states on the pristine GaN nanowires [44], which is consistent with the results of TRPL. In contrast, the OCP of *p*–*n* GaN/CoNiO_x_ nanowires almost fully recovers to its initial level within 80 s after light off, reaching a recovery rate of 94.7%, demonstrating that the CoNiO_x_ decoration might effectively passivate the surface state, eliminate the trapping effect, and facilitate fast carrier transfer. In addition, the SPV was assessed using KPFM. Figure S7 illustrates the CPD of *p*–*n* GaN/CoNiO_x_ nanowires under dark and 340 nm light irradiation, respectively. As shown in Fig. 4c, the SPV of *p*–*n* GaN/CoNiO_x_ nanowires (766.8 mV) was significantly increased by 110.7% compared with the SPV of pristine *p*–*n* GaN nanowires (363.9 mV), indicating that the surface modification effectively enhances the photogenerated carrier separation and allows more photogenerated holes to migrate to the nanowire surface. In short, the TRPL, OCP, and SPV measurements reveal the exceptional capability of CoNiO_x_ as a hole transport layer in facilitating rapid charge extraction. Meanwhile, the possible suppression of carrier trapping could enable rapid carrier migration to the electrolyte to participate in the reaction instead of accumulating on the nanowire surface, thereby improving the photodetection behavior.

The photodetection performance was initially assessed through *i-t* tests (340 nm, 0.1 mW cm^−2^). Figure 4d illustrates a significant enhancement in the photocurrent of *p*–*n* GaN/CoNiO_x_ nanowires (24.8 μA cm^−2^), increasing by 101.6% compared to pristine *p*–*n* GaN nanowires (12.3 μA cm^−2^). Subsequently, the spectral response was assessed by illuminating the samples with light ranging from 265 to 365 nm (Fig. 4e), revealing the improved performance of *p*–*n* GaN/CoNiO_x_ nanowires across the full spectrum. Notably, the *p*–*n* GaN/CoNiO_x_ nanowires exhibit an outstanding responsivity of 247.8 mA W^−1^ at 340 nm. Then, the photocurrent of the *p*–*n* GaN/CoNiO_x_ nanowires was measured under various incident light intensities, as recorded in Fig. S8 and summarized in Fig. 4f. The photocurrent exhibits a near-linear relationship with the increase in light intensity, indicating a great linear photoresponse. The photocurrent of the *p*–*n* GaN/CoNiO_x_ nanowires was measured under different bias voltages using chopped LSV, as recorded in Fig. S9. Photocurrent gradually increased with the potential shift from − 0.15 to 0.55 V vs. Ag/AgCl. This is reasonable because the positive potential accelerates the transfer of holes to the nanowires’ surface. Moreover, surface reactions are more favorable under a more positive bias, ultimately contributing to a larger photocurrent. To unveil the photoresponse speed of the photosensor, the response time (*t*_res_) and recovery time (*t*_rec_) were evaluated and exhibited in Fig. S10. The device demonstrates a fast photoresponse speed with a response time (*t*_res_) of 3 ms and a recovery time (*t*_rec_) of 0.7 ms. Furthermore, the excellent stability of the *p*–*n* GaN/CoNiO_x_ nanowires was demonstrated by the long-term test of the photoelectrode, lasting for 27.5 h (Fig. 4g). Strikingly, no significant attenuation was observed during the test. The insets of Fig. 4g reveal that the photocurrent remains at 90% of its initial value, with a slight decrease from 0.136 to 0.123 μA. Notably, our device is constituted by the PEC photoanode. And the photoanode is usually unstable during operation due to sluggish dynamics and photocorrosion. Here, we demonstrated a stable PEC photoanode by decorating a layer of CoNiO_x_ on nanowires to improve device stability. This remarkable stability can be attributed to the CoNiO_x_ decoration on the nanowires, which acts as a physical protective layer while rapidly consuming the carriers and effectively protecting the nanowires from photocorrosion caused by carrier accumulation [45]. Figure 4h displays the comparison of our device performance with recently reported self-powered PEC-type photosensors, and the responsivity and stability of these devices are summarized in Table 1. Notably, in Table 1, we also summarized and included different GaN nanowires-based photoelectrochemical-type photosensors. Different types of GaN nanowires in the form of heterojunction with other materials have been formed and reported, such as GaN/AlGaN [46], GaN/Pt [47], GaN/Cu [8], GaN/Cu_2_O [48], GaN/AuNC [49], GaN/CsPbBr_3_ [50], and GaN/Ga_2_O_3_ [51]. Impressively, our device exhibits a high responsivity with remarkable device stability which is superior to that of other reported PEC-type photosensors, demonstrating its great potential for future sensitive, sustainable optoelectronic sensing systems.

**Table 1:** Comparison of the performance of various self-powered photoelectrochemical photosensors

Materials	Wavelength (nm)	Responsivity (mA W^−1^)	Stability (h)	Self-powered	Refs
*p*–*n* GaN/CoNiO_x_ nanowires	340	247.8	27.5	Yes	This work
Bi_2_O_2_S nanosheets	365	2.40	1.4	Yes	[52]
a-Ga_2_O_3_ films	254	12.90	0.28	Yes	[53]
Se	525	10.38	2.8	Yes	[54]
2D InSe	365	10.14	0.28	Yes	[55]
Violet phosphorus nanosheets	350	0.03	2.8	Yes	[56]
AlGaN/Rh-Cr_2_O_3_ nanowires	255	175	1.0	Yes	[57]
Pt/AlGaN/GaN	255	37.61	0.97	Yes	[46]
GaN/Pt nanowires	365	42.4	3.39	Yes	[47]
Cu@GaN NWs	458	5.04	0.56	Yes	[8]
GaN@Ga_2_O_3_ NAs	255	93.48	0.17	Yes	[51]
GaN NWs/AuNCs	310	1.9	5.6	Yes	[49]
GaN NWs/CsPbBr_3_	310	1.08	2.8	Yes	[50]
GaN/Cu_2_O NWs	365	0.96	0.14	Yes	[48]

### Demonstration of PEC-type Photosensor for Effective Glucose Detection

**Fig. 5:**
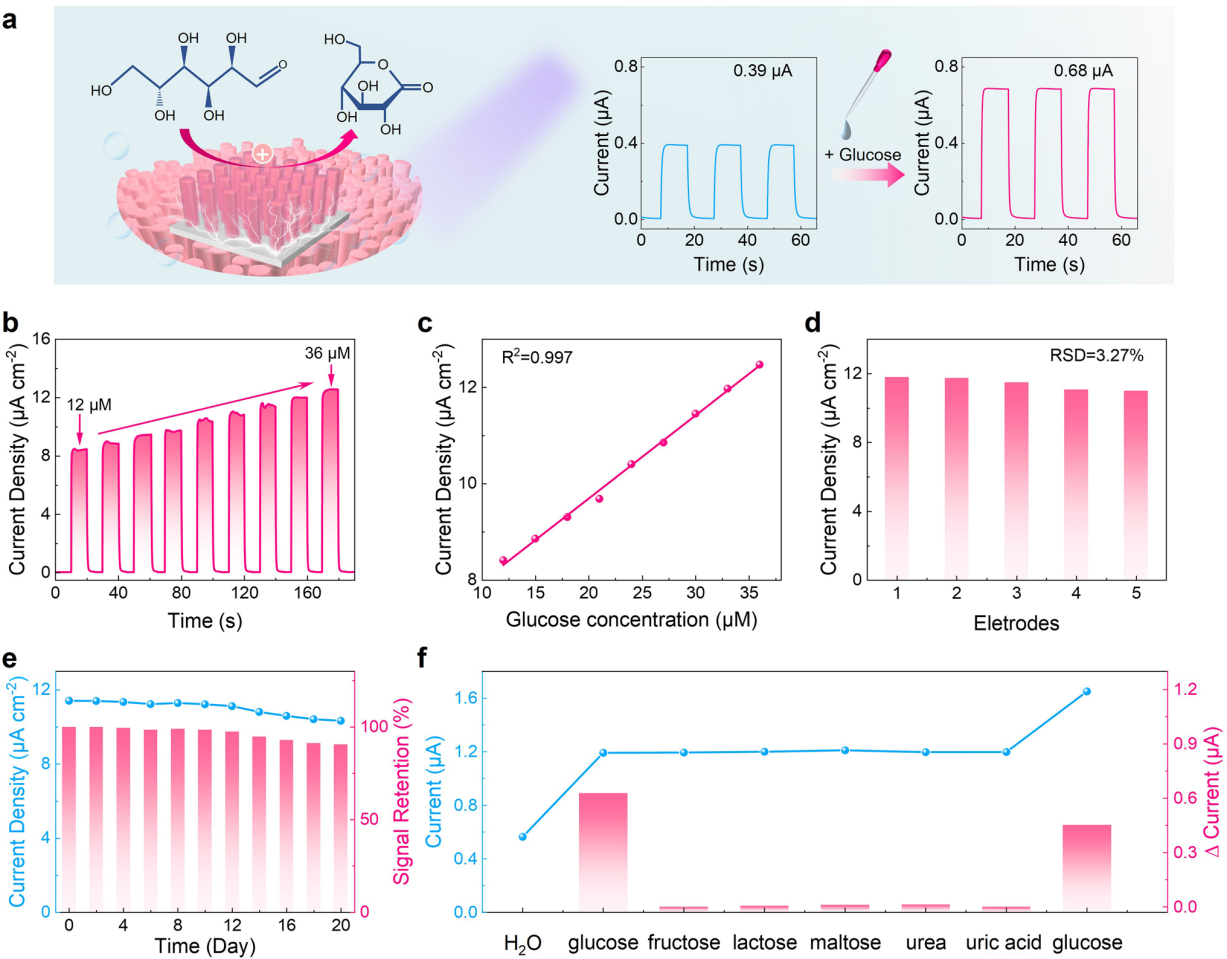
Demonstration of PEC glucose sensing using the *p*–*n* GaN/CoNiO_x_ photoelectrode. **a** Schematic mechanism for PEC glucose sensing using the *p*–*n* GaN/CoNiO_x_ photoelectrode. **b** Photocurrent versus time for the *p*–*n* GaN/CoNiO_x_ photoelectrode with continuous addition of glucose upon 340-nm chopping illumination. **c** The linear relationship between the photocurrent and glucose concentration. **d** Reproducibility tests on five parallel prepared *p*–*n* GaN/CoNiO_x_ photoelectrodes under 30-µM glucose. **e** Stability tests of the *p*–*n* GaN/CoNiO_x_ photoelectrode under 30-µM glucose. The photoelectrode was kept in dry conditions and at room temperature. **f** Effect of interference on the photocurrent by adding fructose, lactose, maltose, urea, and uric acid into the electrolyte

From the above discussion, we have demonstrated the remarkable photodetection performance of the proposed photoelectrode composed of *p*–*n* GaN/CoNiO_x_ nanowires, featuring ultrahigh responsivity and long-term operational stability. In essence, the PEC photodetection process combines conventional semiconductor physical processes with chemical reactions occurring in the electrolyte. These chemical reaction processes encompass the conversion and generation of substances, offering a unique opportunity for the precise identification of chemical and biological substances [21]. Glucose, a fundamental energy source for cells, plays a crucial role in various physiological processes. The accurate and reliable quantitative determination of glucose is of great importance in clinical and biomedical applications [58]. Thus, to demonstrate the practical applicability of the devices, we constructed a PEC glucose sensor based on the *p*–*n* GaN/CoNiO_x_ photoelectrode as a proof-of-concept application, which is schematically depicted in Fig. 5a. Essentially, CoNiO_x_ possesses excellent catalytic properties and serves as a glucose biorecognition unit that may selectively oxidize glucose [59, 60]. We first conducted the measurement using pristine *p*–*n* GaN nanowires. As depicted in Fig. S11, the photocurrent illustrates almost no change by the addition of glucose compared to that observed in the pure electrolyte. Then, we conducted the measurement using *p*–*n* GaN/CoNiO_x_ nanowires (Fig. 5a). Basically, when the photoelectrode was illuminated, electron–hole pairs were generated in the nanowires. Photogenerated holes transferred to the electrolyte and effectively oxidized glucose, converting it into glucolactone [61, 62]. The photoresponse can be enhanced by the addition of glucose compared to that observed in the pure electrolyte (deionized water), confirming the catalytic role of CoNiO_x_ in the glucose detection process. Then, the correlation between glucose concentration and photoresponse can be calibrated, serving as a foundation for the detection of glucose levels.

Figure 5b illustrates the relationship between the photocurrent and the concentration of glucose injected into the electrolyte. Glucose was continuously injected into the electrolyte while the solution was stirred, resulting in an enhancement of the device photoresponse with increasing glucose concentration. A linear correlation was established between the photocurrent and glucose concentration (Fig. 5c), and the regression equation (I in µA cm^−2^ = 6.226 + 0.173 C_glucose_ in µmol L^−1^) was derived. The linear relationship displays a high correlation coefficient (*R*^2^) of 0.997. Notably, the device demonstrates a remarkable sensitivity with a value of 0.173 µA µM^−1^ cm^−2^, along with a low detection limit (LOD) of 0.07 µM (S/N = 3). These results establish a solid foundation for the quantitative determination and detection of glucose. The glucose sensing performance of the *p*–*n* GaN/CoNiO_x_ was compared with those of reported glucose sensors and summarized in Table S2. Compared with other sensors, our device exhibits higher sensitivity and lower detection limit in the detection of glucose. These are attributed to remarkable device stability and excellent glucose catalytic activity. Moreover, compared with most sensors, our device operates under self-powered conditions, which exhibit greater application potential. Moreover, to evaluate the reproducibility, five *p*–*n* GaN/CoNiO_x_ photoelectrodes were prepared using identical conditions, yielding a calculated relative standard deviation (RSD) of 3.27% (Fig. 5d). Additionally, to assess the long-term stability of the PEC sensing system, the constructed photoelectrode was stored under dry conditions at room temperature. The photoresponse was recorded every 2 days, revealing that the photoelectrode maintained 91% of its initial photoresponse over a 20-day storage period (Fig. 5e). In Fig. S12, SEM images exhibit the morphology of the freshly fabricated *p*–*n* GaN/CoNiO_x_ nanowires and the morphology after undergoing 20 days of stability testing. Impressively, the nanowire structure remains nearly unchanged, demonstrating excellent corrosion resistance. Furthermore, we also assessed the selectivity of the glucose sensor. In real glucose sensing conditions, there are other compounds such as fructose, lactose, maltose, urea, and uric acid [63]. The presence of these coexisting compounds can introduce interference and impact the accuracy of glucose sensing. In Fig. S13, we initially added glucose to the electrolyte and observed an increase in current. Subsequently, we sequentially added fructose, lactose, maltose, urea, and uric acid to the solution, but no significant change in current was observed. When glucose was reintroduced to the mixed solution, the current once again exhibited a prominent increase. Figure 5f displays the currents obtained when adding different compounds, as well as the current difference (∆Current) each time a new compound is added (data are extracted from Fig. S13). The ∆Current caused by glucose is significantly greater than those of other interfering compounds at equivalent concentrations. Hence, the interference from these coexisting compounds is negligible, indicating the high selectivity of glucose sensing and verifying the selective catalytic effect of CoNiO_x_ on glucose detection. Finally, the constructed sensor was utilized to examine the glucose levels in human serum. Three human serum samples were obtained, and without any pretreatment, only the serum was diluted in electrolytes before testing. The glucose concentrations measured are shown in Table 2, which are acceptable compared with the values measured at the local hospital. These results provide compelling evidence for the reliability of the fabricated PEC sensor in accurately detecting glucose levels in real human blood serum, demonstrating its practical potential for PEC biosensing applications.

**Table 2:** Determination of glucose concentration in real (human serum) samples

Sample no.	Determined by the hospital (mM)	Determined by the PEC sensor (mM)	Recovery (%)
1	6.41	6.70	104.50
2	8.00	8.45	105.63
3	4.71	5.12	108.68

## Conclusions

Benefiting from the abundant tunability of the PEC device, we have developed a high responsivity and excellent stability photosensor based on *p*–*n* GaN nanowires. The establishment of *p*–*n* junctions within the nanowires significantly enhances the internal carrier separation efficiency of the *p*–*n* GaN nanowires compared to pristine *n*-GaN nanowires. Furthermore, we have strategically designed the surface band bending and then doped the *p*-GaN segment to minimize the hole barrier at the *p*-GaN/electrolyte interface, thereby enhancing the migration of photogenerated holes to the electrolyte. Meanwhile, the reaction activity on the nanowires is enhanced due to the surface CoNiO_x_ modification, which also serves as a hole transport layer to promote charge transfer at the electrode/electrolyte interface. As a result, with the optimization of the internal structure combined with external surface modification, the constructed *p*–*n* GaN/CoNiO_x_ PEC-type photosensors exhibit a high responsivity (247.8 mA W^−1^) and remarkable long-term stability (27.5 h) among all reported PEC-type photosensors. Finally, we constructed a PEC sensing device based on *p*–*n* GaN/CoNiO_x_ photoelectrodes and demonstrated a proof-of-concept glucose sensing application that successfully analyzed blood glucose levels in real human serum samples. Essentially, this work offers a universal route to enhance the efficiency and stability of PEC devices, ensuring their overall performance and inspiring applications in the future biosensing and detection fields.

## Supplementary Information

Below is the link to the electronic supplementary material.

Supplementary file1 (DOCX 3556 kb)
