# Supplementary material for: Facile Semiconductor p–n Homojunction Nanowires with Strategic p-Type Doping Engineering Combined with Surface Reconstruction for Biosensing Applications
Source: Nanomicro Lett. 2024 May 14;16:192. doi: 10.1007/s40820-024-01394-5 (PMC11093954; doi:10.1007/s40820-024-01394-5)
Supplement: Supplementary file 1 — Supplementary file1 (DOCX 3556 kb) [file 40820_2024_1394_MOESM1_ESM.docx]

Supporting Information for

**Facile Semiconductor *p*-*n* Homojunction Nanowires with Strategic *p*-Type Doping Engineering Combined with Surface Reconstruction for Biosensing Applications**

Liuan Li^1,^†, Shi Fang^1,^†, Wei Chen^1^, Yueyue Li^2^, Mohammad Fazel Vafadar^3^, Danhao Wang^1^, Yang Kang^1^, Xin Liu^1^, Yuanmin Luo^1^, Kun Liang^1^, Yiping Dang^4^, Lei Zhao^4^, Songrui Zhao^3^, Zongzhi Yin^2,^*, Haiding Sun^1,^*

^1^ iGaN Laboratory, School of Microelectronics, University of Science and Technology of China, Hefei 230026, P. R. China

^2^ Department of Obstetrics and Gynecology, the First Affiliated Hospital of Anhui Medical University, No 218 Jixi Road, Hefei 230022, P. R. China

^3^ Department of Electrical and Computer Engineering, McGill University, 3480 University Street, Montreal, Quebec H3A 0E9, Canada

^4^ Union Hospital, Tongji Medical College, Huazhong University of Science and Technology, No 1277 Jiefang Ave. Wuhan 430022, P. R. China

† Liuan Li and Shi Fang contributed equally to this work.

*Corresponding authors. E-mail: [yinzongzhi@ahmu.edu.cn](mailto:yinzongzhi@ahmu.edu.cn) (Zongzhi Yin); [haiding@ustc.edu.cn](mailto:haiding@ustc.edu.cn) (Haiding Sun)

**Supplementary Figures and Tables**

**
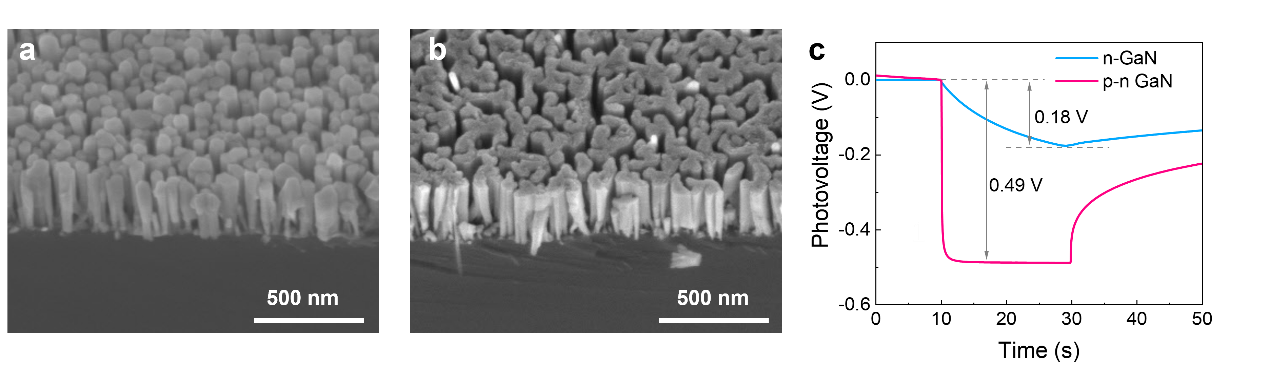
**

**Fig. S1** Scanning electron microscope (SEM) images of **a** *n*-GaN and **b** *p*-*n* GaN nanowires. **c** Open-circuit potential (OCP) measurements of the *n*-GaN and *p*-*n* GaN nanowires under 340 nm light illumination. The ΔOCP in *p*-*n* GaN nanowires significantly exceeds that in *n*-GaN nanowires, providing evidence for the superior carrier separation and extraction efficiency within *p*-*n* GaN nanowires compared to *n*-GaN nanowires.

**
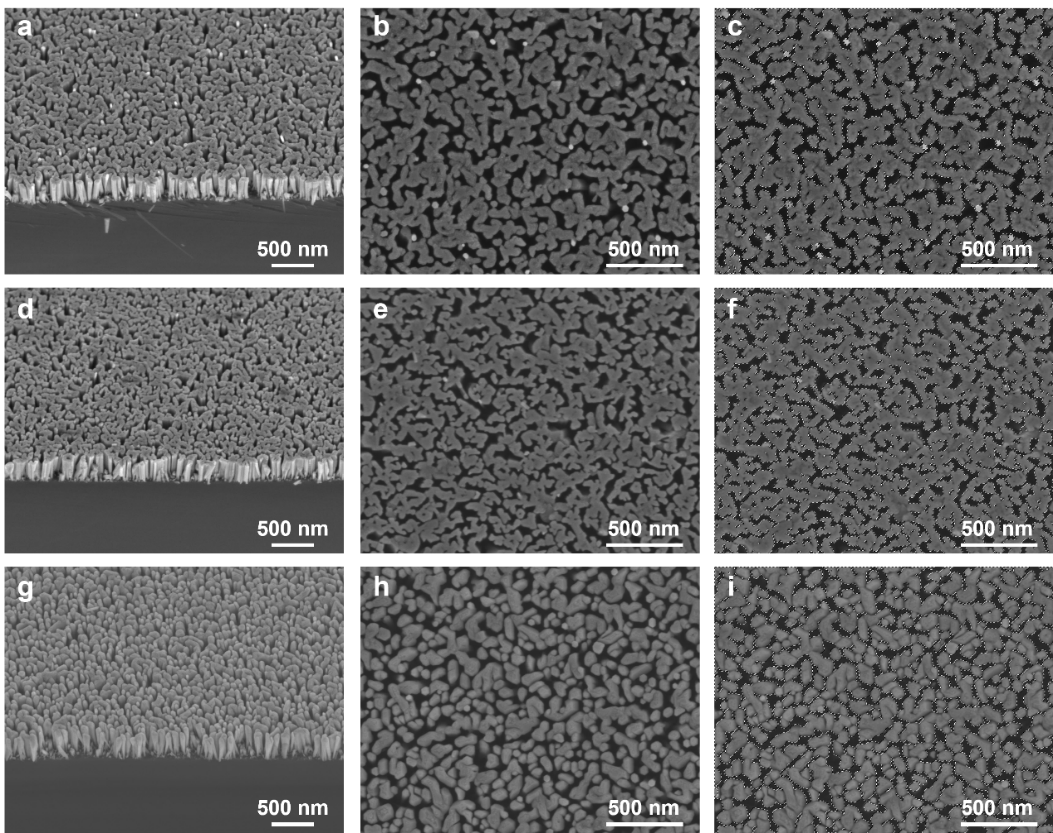
**

**Fig. S2 a** Side-view and **b** top-view SEM images of the *p*-*n* GaN nanowires, **c** the corresponding selected nanowires area of the *p*-*n* GaN nanowires. The filling factor is approximately 0.76. **d** Side-view and **e** top-view SEM images of the *p*^+^-*n* GaN nanowires, **f** the corresponding selected nanowires area of the *p*^+^-*n* GaN nanowires. The filling factor is approximately 0.77. **g** Side-view and **h** top-view SEM images of the *p*^++^-*n* GaN nanowires, **i** the corresponding selected nanowires area of the *p*^++^-*n* GaN nanowires. The filling factor is approximately 0.80. The filling factors are calculated by the ratio of the nanowires area to the whole image area.


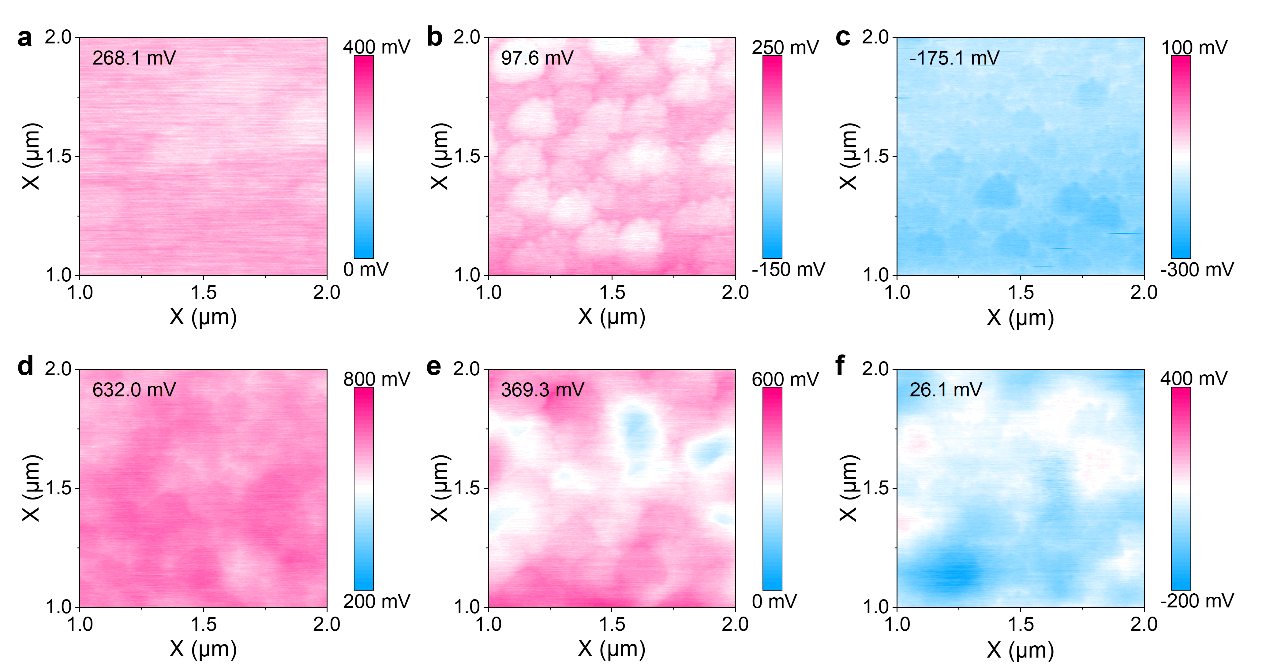


**Fig. S3** Contact potential difference (CPD) mappings were obtained under dark conditions for **a** *p*-*n* GaN, **b** *p*^+^-*n* GaN, and **c** *p*^++^-*n* GaN nanowires, respectively. The corresponding average CPD values in a 2 μm × 2 μm region of *p*-*n* GaN, *p*^+^-*n* GaN, and *p*^++^-*n* GaN nanowires are 268.1, 97.6, and -175.1 mV, respectively. Additionally, CPD mappings were recorded under the illumination of 340 nm for **d** *p*-*n* GaN, **e** *p*^+^-*n* GaN, and **f** *p*^++^-*n* GaN nanowires, respectively. The corresponding average CPD values in a 2 μm × 2 μm region of *p*-*n* GaN, *p*^+^-*n* GaN, and *p*^++^-*n* GaN nanowires are 632.0, 369.3, and 26.1 mV, respectively. The difference value between the contact potential under illuminated conditions and dark conditions corresponds to surface photovoltage (SPV). The calculated SPV values of *p*-*n* GaN, *p*^+^-*n* GaN, and *p*^++^-*n* GaN nanowires are 363.9, 271.7, and 201.2 mV, respectively.

**Fig. S4** valence band spectra of the **a** *p*-*n* GaN, **b** *p*^+^-*n* GaN, and **c** *p*^++^-*n* GaN nanowires. The relative positions of the valence band maxima referred to the Fermi level in *p*-*n* GaN, *p*^+^-*n* GaN, and *p*^++^-*n* GaN nanowires were determined as 1.53, 1.36, and 1.12 eV, respectively.


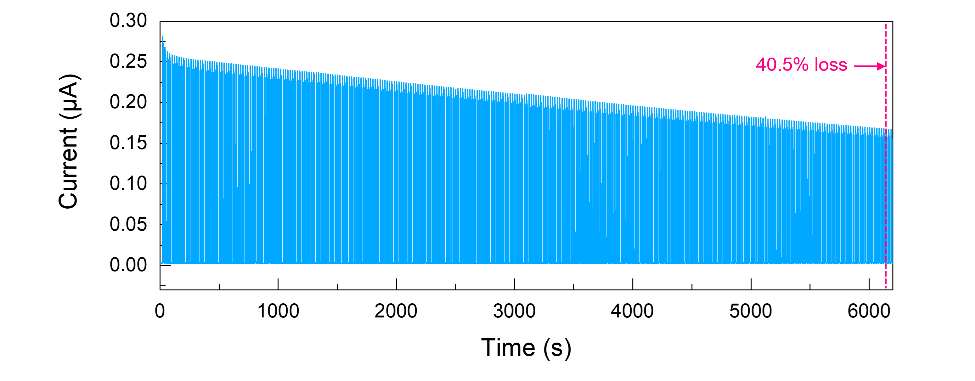


**Fig. S5** The continuous on/off cycles test of *p*-*n* GaN nanowires under the illumination of 340 nm. The light intensity is kept at 0.1 mW cm^−2^.


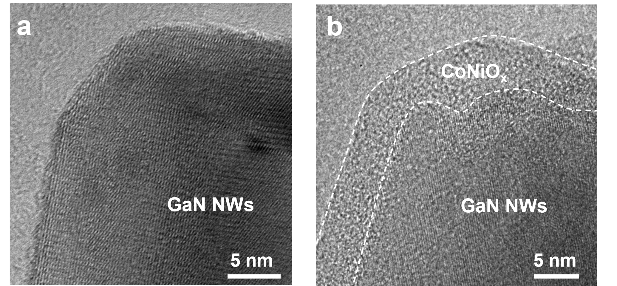


**Fig. S6** High-resolution TEM image of the **a** *p*-*n* GaN nanowires and **b** *p*-*n* GaN/CoNiO_x_ nanowires. We can clearly observe the periodic lattice fringes in the GaN nanowires as well as the coated amorphous layer around the nanowires surface.


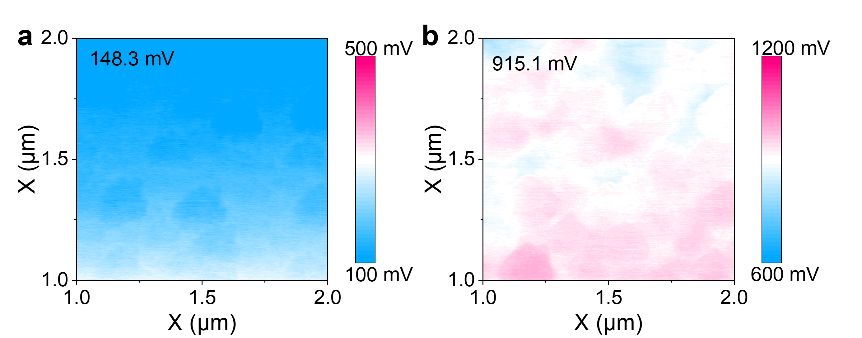


**Fig. S7** The CPD mappings of *p*-*n* GaN/CoNiO_x_ nanowires under **a** dark conditions and **b** under the illumination of 340 nm. The calculated SPV values of *p*-*n* GaN nanowire/CoNiO_x_ nanowires were 766.8 mV.


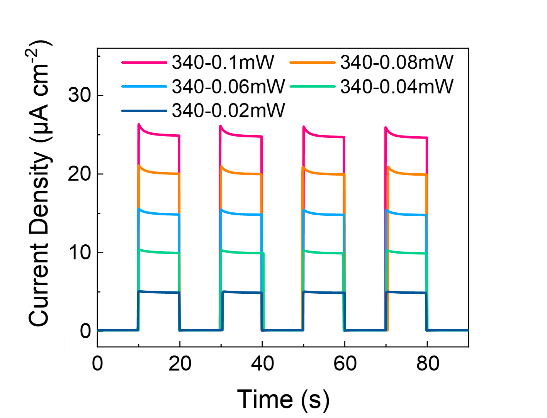


**Fig. S8** Photocurrents of the *p*-*n* GaN/CoNiO_x_ nanowires under the illumination of 340 nm with different light intensities


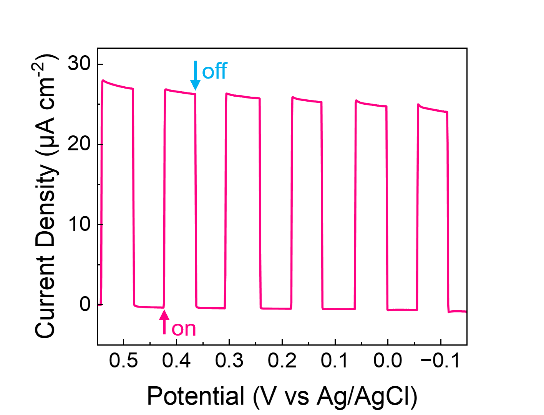


**Fig. S9** Chopped LSV measurements from 0.55 to −0.15 V with a scan rate of 0.03 V s^−1^


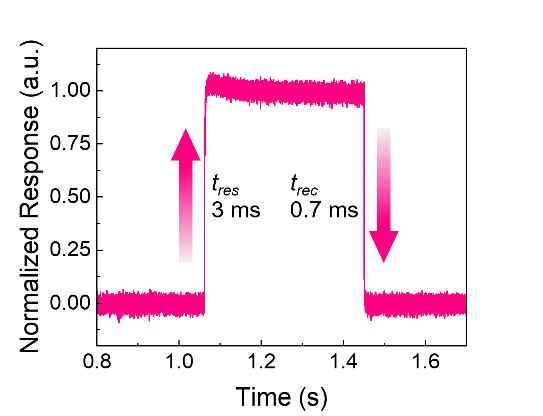


**Fig. S10** Analysis of the response/recovery times of PEC photosensor employing *p*-*n* GaN/CoNiO_x_


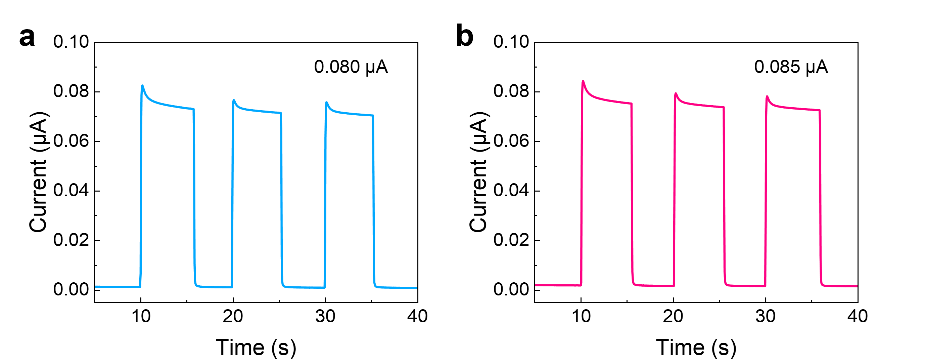


**Fig. S****11** Photocurrents of the *p*-*n* GaN nanowires measured under **a** pure electrolyte (deionized water) and **b** 30 µM glucose. The photocurrent measured under pure electrolyte and 30 µM glucose is 0.080 and 0.085 µA, respectively.


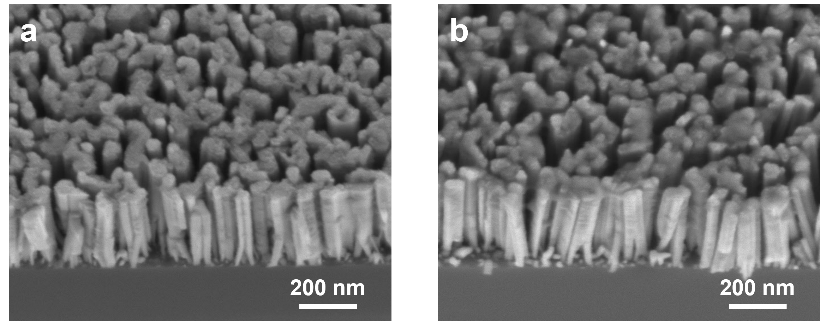


**Fig. S12** SEM images exhibit the morphology of the p–n GaN/CoNiO_x_ nanowires **a** before and **b** after the 20-day test.


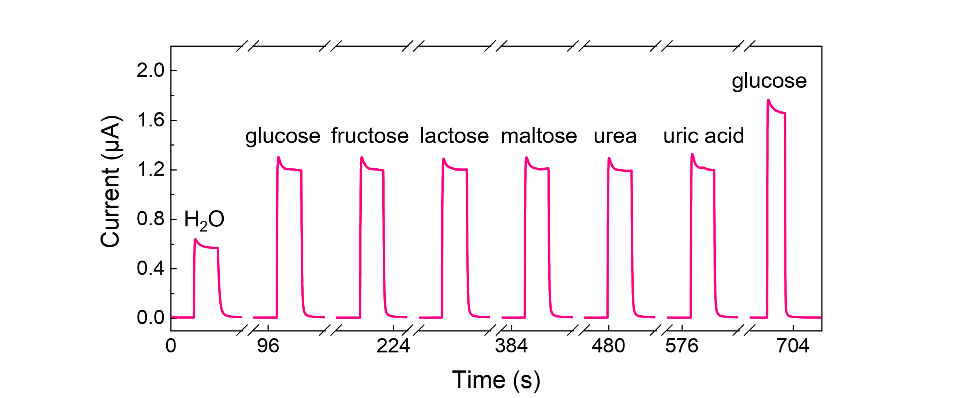


**Fig. S****13** Sequential additions of glucose, fructose, lactose, maltose, urea, and uric acid were made to the electrolyte under chopper light, and the corresponding photocurrents were recorded. The concentrations of all these compounds are 50µM. Two additions of glucose caused a significant enhancement of the photocurrent, while the intermediate addition of interfering substances showed negligible changes in the photocurrent. These results indicate the high selectivity of the glucose sensing.


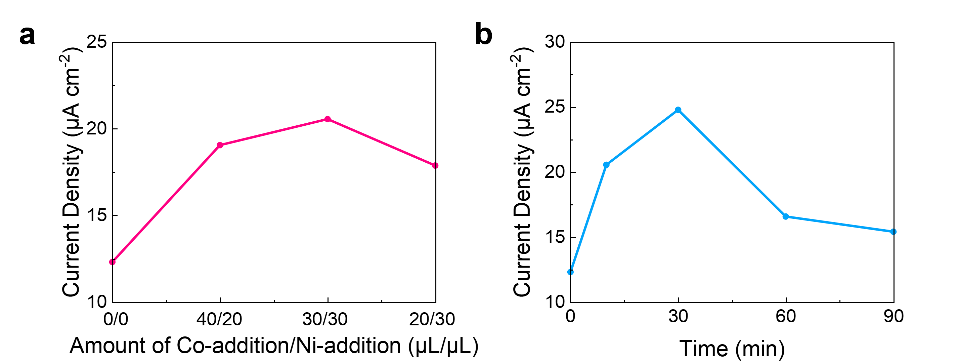


**Fig. S14** **a** Relationship between the photoresponse and the Co/Ni loading amounts. The photodeposition time is kept as 10 minutes. **b** Relationship between the photoresponse and the photodeposition time. The amount of Co-addition/Ni-addition is 30/30 µL. It is found that the 30 µL Co-addition, 30 µL Ni-addition, and the photodeposition for 30 minutes is the optimal photodeposition condition.


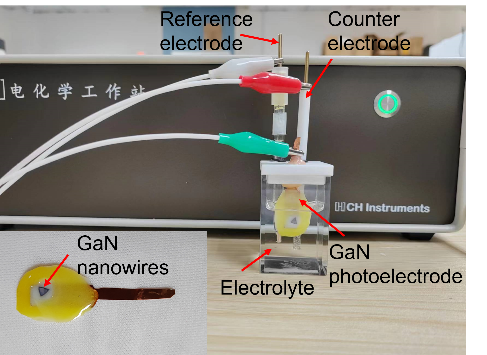


**Fig. S15** Image of the as-fabricated PEC photosensor. The inset shows the fabricated GaN nanowires photoelectrode.

**Table S1** Fitted parameters for the TRPL curves of *p*-*n* GaN and *p*-*n* GaN/CoNiO_x_ nanowires

| Sample | τ_1_ (ns) | % | τ_2_ (ns) | % | τ_av_ (ns) |
| --- | --- | --- | --- | --- | --- |
| *p*-*n* GaN | 0.144 | 78.1 | 1.253 | 21.9 | 0.387 |
| *p*-*n* GaN/CoNiO_x_ | 0.239 | 54.3 | 1.630 | 45.7 | 0.875 |

**Table S2** Comparison of the performance of various glucose sensors

| Materials | Potential | Detection limit (µM) | Sensitivity  (µA mM^-1^ cm^-2^) | Refs. |
| --- | --- | --- | --- | --- |
| *p*-*n* GaN/CoNiO_x_ nanowires | 0 V vs. Ag/AgCl | 0.07 | 173 | This work |
| ITO/TiO_2_- Co_3_O_4_-CNT-GO_x_ | 0 V vs. Ag/AgCl | 0.16 | 0.3 | [S1] |
| Ni/nanodiamond/BDD | 0.46 V vs. SCE | 0.05 | 120 | [S2] |
| BiOCl-G NHS | 0.5 V vs. Ag/AgCl | 220 | 127.2 | [S3] |
| Cu/ZnO | 0.1 V vs. SCE | 3.762 | 63.76 | [S4] |
| CuO/FTO | 0.6 vs. Ag/AgCl | 59.5 | 263 | [S5] |
| Au/Ni/BDD | 0.5 V vs. Ag/AgCl | 2.6 | 157.5 | [S6] |
| 3D hollow-out TiO_2_ NWc/GO_x_ | 0.4 V vs. Ag/AgCl | 8.7 | 58.9 | [S7] |
| NiAl-LDH/α-Fe_2_O_3_ | 0.3 V vs. SCE | 5 | 274.7 | [S8] |
| Fe_2_O_3_NR/FTO | -0.1 V vs. SCE | 5.5 | 100.46 | [S9] |
| Fe_2_O_3_ films | 0.99 V vs. RHE | 0.05 | 17.23 | [S10] |

SCE: Saturated calomel electrode; RHE: Reversible hydrogen electrode

**Supplementary References**

1. B. Çakıroğlu, M. Özacar, A self-powered photoelectrochemical glucose biosensor based on supercapacitor Co_3_O_4_-CNT hybrid on TiO_2_. Biosens. Bioelectron. **119**, 34-41 (2018). <https://doi.org/10.1016/j.bios.2018.07.049>
2. W. Dai, M. Li, S. Gao, H. Li, C. Li et al., Fabrication of nickel/nanodiamond/boron-doped diamond electrode for non-enzymatic glucose biosensor. Electrochim. Acta. **187**, 413-421 (2016). <https://doi.org/10.1016/j.electacta.2015.11.085>
3. A. I. Gopalan, N. Muthuchamy, K. P. Lee, A novel bismuth oxychloride-graphene hybrid nanosheets based non-enzymatic photoelectrochemical glucose sensing platform for high performances. Biosens. Bioelectron. **89**, 352-360 (2017). <https://doi.org/10.1016/j.bios.2016.07.017>
4. B. Yang, N. Han, S. Hu, L. Zhang, S. Yi et al., Cu/ZnO nano-thorn with modifiable morphology for photoelectrochemical detection of glucose. J. Electrochem. Soc. **168**, 027516 (2021). <https://doi.org/10.1149/1945-7111/abe50e>
5. N. J. Cory, E. Visser, J. Chamier, J. Sackey, F. Cummings et al., Electrodeposited CuO thin film for wide linear range photoelectrochemical glucose sensing. Appl. Surf. Sci. **576**, 151822 (2022). <https://doi.org/10.1016/j.apsusc.2021.151822>
6. K. Yao, B. Dai, X. Tan, V. Ralchenko, L. Yang et al., Fabrication of Au/Ni/boron-doped diamond electrodes via hydrogen plasma etching graphite and amorphous boron for efficient non-enzymatic sensing of glucose. J. Electroanal. Chem. **871**, 114264 (2020). <https://doi.org/10.1016/j.jelechem.2020.114264>
7. W. Yang, X. Wang, W. Hao, Q. Wu, J. Peng et al., 3d hollow-out TiO_2_ nanowire cluster/GO_x_ as an ultrasensitive photoelectrochemical glucose biosensor. J. Mater. Chem. B. **8**, 2363-2370 (2020). <https://doi.org/10.1039/d0tb00082e>
8. M. Fan, S. Zhu, Q. Zhang, X. Wang, L. Zhang et al., Sensitive photoelectrochemical sensing of glucose using hematite decorated with NiAl-layered double hydroxides. Food Chem. **405**, 134883 (2023). <https://doi.org/10.1016/j.foodchem.2022.134883>
9. L. He, Q. Zhang, C. Gong, H. Liu, F. Hu et al., The dual-function of hematite-based photoelectrochemical sensor for solar-to-electricity conversion and self-powered glucose detection. Sens. Actuators, B **310**, 127842 (2020). <https://doi.org/10.1016/j.snb.2020.127842>
10. F. Liu, P. Wang, Q. Zhang, Z. Wang, Y. Liu et al., α‐Fe_2_O_3_ film with highly photoactivity for non‐enzymatic photoelectrochemical detection of glucose. Electroanalysis. **31**, 1809-1814 (2019). <https://doi.org/10.1002/elan.201900133>
